# Two-dimensional imaging in hyperbolic media–the role of field components and ordinary waves

**DOI:** 10.1038/srep17690

**Published:** 2015-12-04

**Authors:** Alessandro Tuniz, Boris T. Kuhlmey

**Affiliations:** 1 Institute of Photonics and Optical Science (IPOS), School of Physics, University of Sydney, NSW, 2006, Australia; 2 Centre for Ultrahigh bandwidth Devices for Optical Systems (CUDOS), University of Sydney, NSW 2006, Australia; 3 Leibniz Institute of Photonic Technology (IPHT Jena), Albert-Einstein-Str. 9, 07745 Jena, Germany

## Abstract

We study full vector imaging of two dimensional source fields through finite slabs of media with extreme anisotropy, such as hyperbolic media. For this, we adapt the exact transfer matrix method for uniaxial media to calculate the two dimensional transfer functions and point spread functions for arbitrary vector fields described in Cartesian coordinates. This is more convenient for imaging simulations than the use of the natural, propagation direction-dependent TE/TM basis, and clarifies which field components contribute to sub-diffraction imaging. We study the effect of ordinary waves on image quality, which previous one-dimensional approaches could not consider. Perfect sub-diffraction imaging can be achieved if longitudinal fields are measured, but in the more common case where field intensities or transverse fields are measured, ordinary waves cause artefacts. These become more prevalent when attempting to image large objects with high resolution. We discuss implications for curved hyperbolic imaging geometries such as hyperlenses.

Materials with extreme anisotropy, and in particular indefinite materials such as metal/dielectric multilayer and wire array metamaterials, can beat the diffraction limit because they carry propagating extraordinary waves with high spatial frequencies that are evanescent in conventional dielectrics^1^. For extreme anisotropy, all extraordinary waves have similar phase velocity, so that slabs of such materials are sub-diffraction endoscopes^2^. Magnifying or focusing hyperlenses can be obtained from curved geometries^3^,^4^. Experimental implementations at microwave, terahertz, visible and ultra-violet frequencies have been demonstrated^5^,^6^,^7^,^8^,^9^,^10^, with numerous potential applications. However, in spite of the fact that hyperbolic media are able to carry high spatial frequencies, images obtained through hyperlenses and hyperbolic media slabs are not perfect. Known sources of artefacts include chromatic dispersion and resonances of slab waves, that can add minor side-lobes (at best) or completely scramble images (at worst), and diffracting ordinary waves which add unwanted blurriness and background noise^5^,^11^,^12^,^13^,^14^,^15^. While these artefacts are known or at least expected, a deeper understanding of their impact, and how to avoid or correct them, is of particular importance for future applications of hyperbolic media slabs and hyperlenses.

Numerical and theoretical studies of imaging through slabs of extremely anisotropic media, and of artefacts in particular, have so far either relied on full 3D numerical simulations such as finite element methods^12^, or considered the 2D problem of propagation along a longitudinal dimension of spatial frequencies in one transverse dimension only^13^,^14^,^16^. General three dimensional numerical methods can be slow and can have convergence problems, in particular if one of the permittivity component is large or negative. Reducing the problem to two dimensions simplifies the use of transfer matrix methods, as ordinary waves decouple form extraordinary waves. Indeed, this has yielded considerable insight in artefacts due to extraordinary waves only^14^,^15^. However this decoupling also means that the role of unwanted ordinary wave excitation in imaging cannot be studied using two dimensional methods: In three dimensions, even for linearly polarized light, both ordinary and extraordinary waves are excited in a non trivial manner, affecting imaging. Here we apply the well known exact transfer matrix method (TMM) to calculate the full three-dimensional propagation of fields through finite slabs of extreme uniaxial media. In contrast to previous studies using TMM, we consider two dimensional images from any object, represented by a known field in Cartesian coordinates. A related approach using Hankel transforms and one dimensional transfer matrix methods was used by Kotyński *et al.*^17^ in the context of dielectric/metallic multilayers. However in that work the longitudinal cross coupling was ignored, which, as we show here, becomes of particular importance for near field imaging where it enables the extraction of perfect images.

We then use this method to analyse imaging transfer functions and two-dimensional point spread functions, as well as imaging artefacts due to ordinary waves, and present strategies to avoid them. The method lends itself to rapid exact calculation of images through slabs of uniaxial materials, and can be extended to exact calculations of images through planar layered hyperbolic media stacks as well as non-local models for wire media.

## Results

### Method

**Figure 1:**
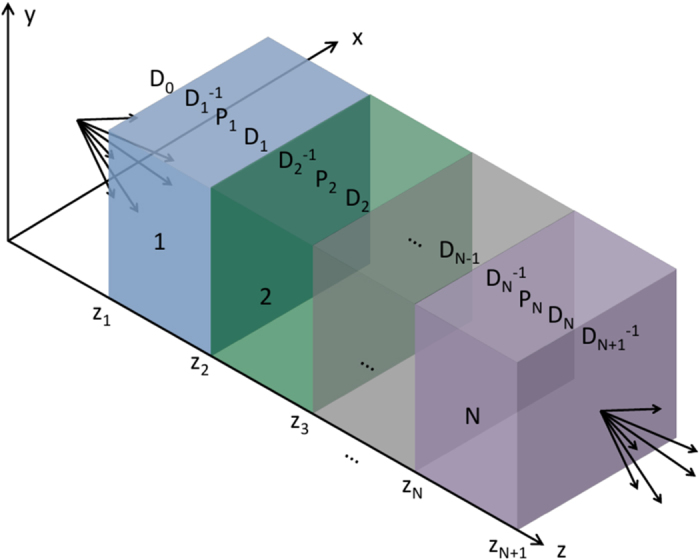
Schematic of the general geometry under consideration, for finding transfer matrices of uniaxial materials in the TE/TM bases.

We consider a slab of uniaxial material, or more generally a multilayer stack of such materials (Fig. 1). Our aim is to calculate the transfer of an image, as described by a *x*- and *y*- dependent time harmonic field in a given plane *z*, through a uniaxial slab of finite thickness using the TMM. Although the TMM for uniaxial planar slabs has been solved decades ago using transfer matrices and can be found in the literature^16^,^18^, it relies on closed form expression of dynamic and propagation matrices in the natural TE/TM basis for given transverse wavevectors. Therefore, in order to use this method, a given image described as a field distribution in two-dimensional Cartesian coordinates must be expressed in the basis of TE and TM modes. First a spatial Fourier transform along *x* and *y* of the vector field components of the image is taken at the input; for each spatial frequency set 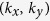, fields are then projected on the TE/TM basis in which transfer matrices are expressed; the TMM method is then used to propagate all spatial frequencies; finally, the inverse transform is used to reconstruct the fields at the other end. Here, we present the complete formalism of this method explicitly, which can be used to calculate images as produced by a uniaxial slab (and stacks of such slabs) for any arbitrary input field distribution.

We first introduce all notations and general characteristics of waves in homogeneous uniaxial media required for the derivations. We then introduce the boundary conditions and propagation matrices to build the usual transfer matrix method, and introduce the projection matrices required to apply the transfer matrix method to arbitrary field distributions. The method is finally used to explore imaging artefacts in hyperbolic media slabs. We also discuss how far our findings apply to curved geometries such as hyperlenses.

#### Basis of TE and TM waves in uniaxial media

The first step in the method is to determine the wavevector-dependent TE and TM waves in which the TMM matrices are expressed. We consider harmonic plane waves with wave vector **k**, and angular frequency *ω*, in a non-magnetic (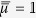), homogeneous medium with relative permittivity tensor in each layer


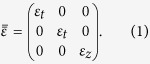


The wave equation for the plane waves becomes


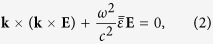


which can then be written explicitly as


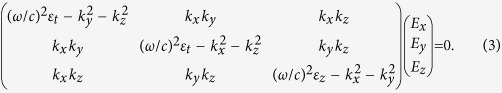


For nontrivial plane wave solutions to exist, the determinant of the matrix of Eq. (3) must vanish, yielding a quartic equation for 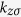 with four roots σ = 1, 2, 3, 4,


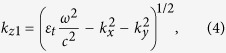



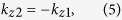



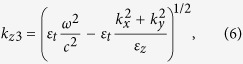



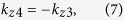


corresponding to the forward- and backward-propagating TE, and forward- and backward-propagating TM modes, respectively. In an uniaxial medium, the TE waves are the ordinary waves, and TM waves the extraordinary waves. Fields with spatial frequency 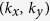 in the medium must be a superposition of such modes, with electric fields given by


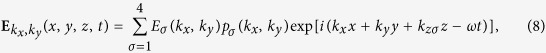


where 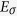 are expansion coefficients, and 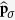 are complex polarization unit vectors given by


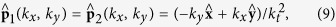



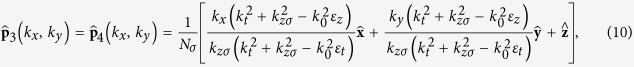


where *N*_*σ*_ are normalization constants such that 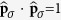. Maxwell’s equations yield the associated magnetic fields,


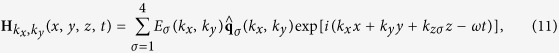


where


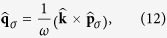


which are not generally unit vectors.

## Transfer matrices in TE/TM basis

In view of building the transfer matrix method, we now consider the boundary conditions for fields of given 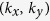 at interfaces between uniaxial media with aligned optical axis *z* orthogonal to their interface, as shown in Fig. 1. We consider the general case of multiple uniaxial layers, and express electric and magnetic fields **E** and **H** in layer *n* as


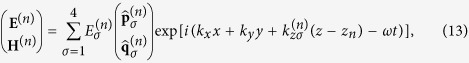


where *z*_*n*_ is the *starting* position of the 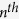 layer (for the *n* = 0 layer, corresponding to the half space to the left of the stack, *z*_0_ can be chosen arbitrarily as the phase reference, but is most conveniently set to be *z*_0_ = *z*_1_). Imposing the boundary conditions of continuity of the tangential components of **E** and **H** at a boundary 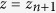 results in


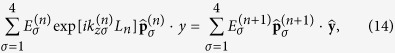



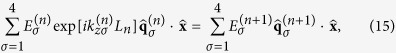



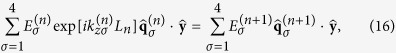



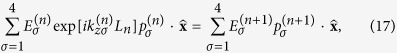


where 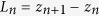 is the thickness of the *n*^*th*^ layer. This can be re-written in compact form:


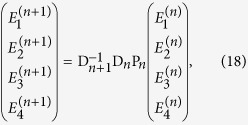


where we introduce the dynamic matrix D*_n_* for medium *n*


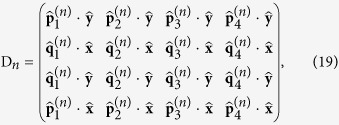


and the propagation matrix P_*n*_ for layer *n*


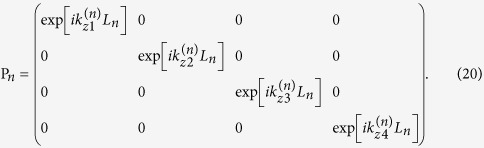


By applying Eq. (18) iteratively, it is possible to relate the field coefficients at the output to those at the input for an arbitrary stack. We emphasize that all above matrices and bases 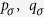 depend on *k*_*x*_ and *k*_*y*_. The transmission matrix of the stack can then be defined as


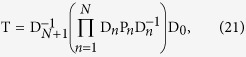


for which we then have


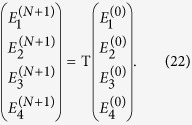


## Specific case — single uniaxial slab

**Figure 2:**
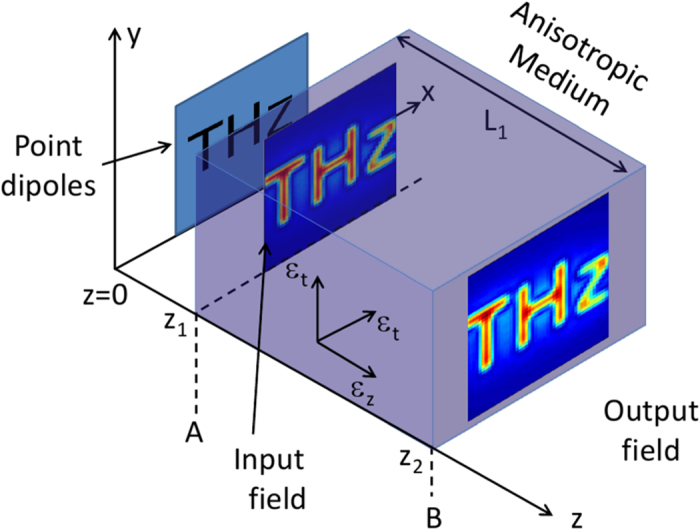
Schematic of the specific case under consideration. Sources (point dipoles) at a position *z* = 0 generate an image at input *A* (*z* = *z*_1_) of a single uniaxial slab of thickness *L*_1_ = *z*_2_−*z*_1_. The output fields at the slab output *B* (*z* = *z*_2_) are calculated as in the text.

A number of additional steps are required to use the transmission matrix to calculate images and point spread functions. For simplicity, and without loss of generality, we now consider a single anisotropic dielectric slab of thickness *L*_1_ in air, as shown in the schematic of Fig. 2. We relate the coefficients at the output boundary (point B, noting the same coefficients describe the fields for all 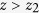 through Eqs. (8) and (11)) to the coefficients at the input boundary (point A, noting again that the same coefficients describe the fields for all 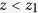) via the transfer matrix T. In the absence of backward propagating waves for 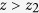 we have 
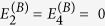
, so that we may write:


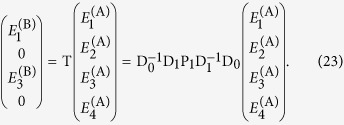


If propagation through a stack of slabs rather than a single slab is to be considered, T can simply be replaced by its more general form Eq. (21). Note that we have used the fact that the first and last layers are both air, so that D_2_ = D_0_.

More conveniently, we wish to relate the *incident* field coefficients to the *transmitted* and *reflected* coefficients, using a 4 × 4 scattering matrix S:


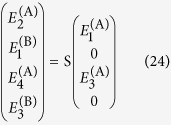


Equations (23)–(24), 40 can be solved simultaneously to relate the calculated elements of T with those of S, providing the fields reflected and transmitted by the slab for a given input. Explicit expressions for the general case are contained in the Supplementary Methods 1.1.

Fields in each layer have so far been expressed in a four-dimensional orthogonal basis 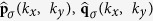 such that, for a given 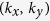, they are composed of forward- and backward- propagating TE and TM modes with fields expressed as in Eq. (13). In practice, electromagnetic fields at the input and output are more conveniently expressed in a three-dimensional orthonormal Cartesian basis 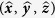 such that 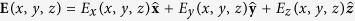 and 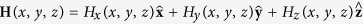. Taking the 2D-Fourier transform at a certain *z* results in 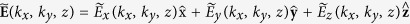 and 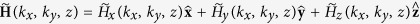. Equating these expressions at *z* = *z*_*n*_ to the fields as expressed in Eq. (13) we can write,


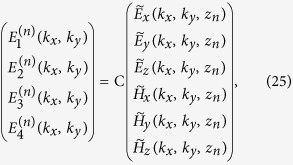


where C is a 4 × 6 change of basis matrix depending on 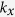 and 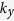 and the layer, that can be determined by taking dot products of the different expressions with 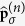 and 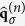. In isotropic layers - as is the case for air at the input and output of the slab– Eqs. (9), (10), (12) lead to equality and orthogonality relations that can be used to express C explicitly:


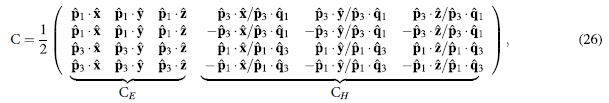


where C*_E_* and C*_H_* are 4 × 3 matrices that relate electric- and magnetic- field contributions to 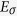, respectively, and we omit the ^(*n*)^ superscript for the basis vectors of each layer to simplify notations. Correspondingly, we may write


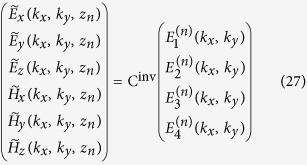


where we have defined the “inverse” change of basis matrix C^inv^ such that


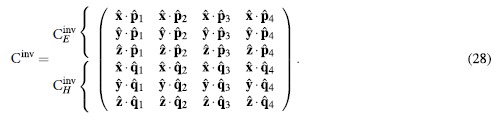



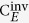
 and 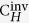 are 3 × 4 matrices that express how 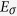 coefficients contribute to the electric and magnetic fields, respectively, at 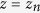. Equations (27) and (30) simply express Eqs. (8) and (11), and unlike Eq. (26) do not rely on the special cases of relations Eqs. (9) and (10), and is also valid in anisotropic layers.

Note that while for a given set of 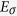 coefficients, C^inv^ determines uniquely all field components in Cartesian coordinates, the converse is not true: arbitrary functions expressed in Cartesian coordinates are not necessarily a unique superposition of TE and TM modes, since not all functions of space satisfy Maxwell’s equations. Two arbitrary functions of space may thus be mapped to the same set of 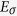 coefficients through Eq. (26). However any field satisfying Maxwell’s equations will be mapped to itself through multiplication by C^inv^C. For an arbitrary field with components propagating in both positive and negative 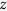 directions, it is necessary to use the complete **E** and **H** fields with the full 4 × 6 matrix C in Eq. (26). Indeed, an identical **E** distribution determined on a single *z* plane can propagate in either 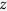 direction; the direction of propagation is then determined through the relative sign of the **H** components. However, since for use in Eq. (24) we only need 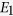 and 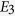 which are *known* to propagate in the positive *z* direction only, Eq. (26) can be simplified to express 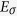 in terms of C_*E*_ and the electric field only–setting the 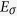 coefficients corresponding to backward- or forward- propagation respectively to zero. This is the case when calculating the transmission of images generated from objects consisting of sources exclusively on the input side of the slab. The formalism then can also provide the reflected field back to the sources from the slab.

Explicitly, for fields radiating from sources exclusively at positions 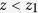, combining Eqs. (24, 25, 26, 27) the (Cartesian) transfer matrix then relates the transmitted field to the input field, both expressed in Cartesian coordinates:


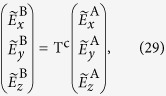


where the 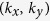-dependent transfer matrix T^c^ for Cartesian fields is defined as


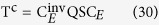


with


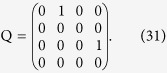


The matrix Q simply enforces the condition that fields are incoming from 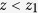 and extracts the transmitted fields from the vector in the left hand side of Eq. (24) to the correct basis for 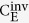. It is important to note that the matrix T^c^ has no reason to be diagonal. In general the transmission of electric fields and magnetic fields are coupled, so that the output electric field also depends on the input magnetic field. It may thus be surprising that in Eq. (31) we can express the transmitted electric field solely in terms of the incoming electric field. To understand this it is important to recall that in Eq. (31) 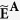 is not the total field on side A, but only the incoming part of the field, which is the field generated by sources at 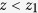 calculated without reflection from the interface (i.e. calculated in free space). For such a forward propagating field in a non magnetic medium (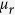 isotropic and unity), the magnetic field is directly given by the electric field through Maxwell-Faraday law for a forward propagating plane wave 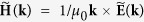. The forward propagating incoming field can thus be entirely described by the electric field only. The total electric field at the input face also includes backward waves due to reflection from the slab, which one can obtain from Eq. (24) from the incident field.

## Application to imaging

### Cartesian transfer matrix

**Figure 3:**
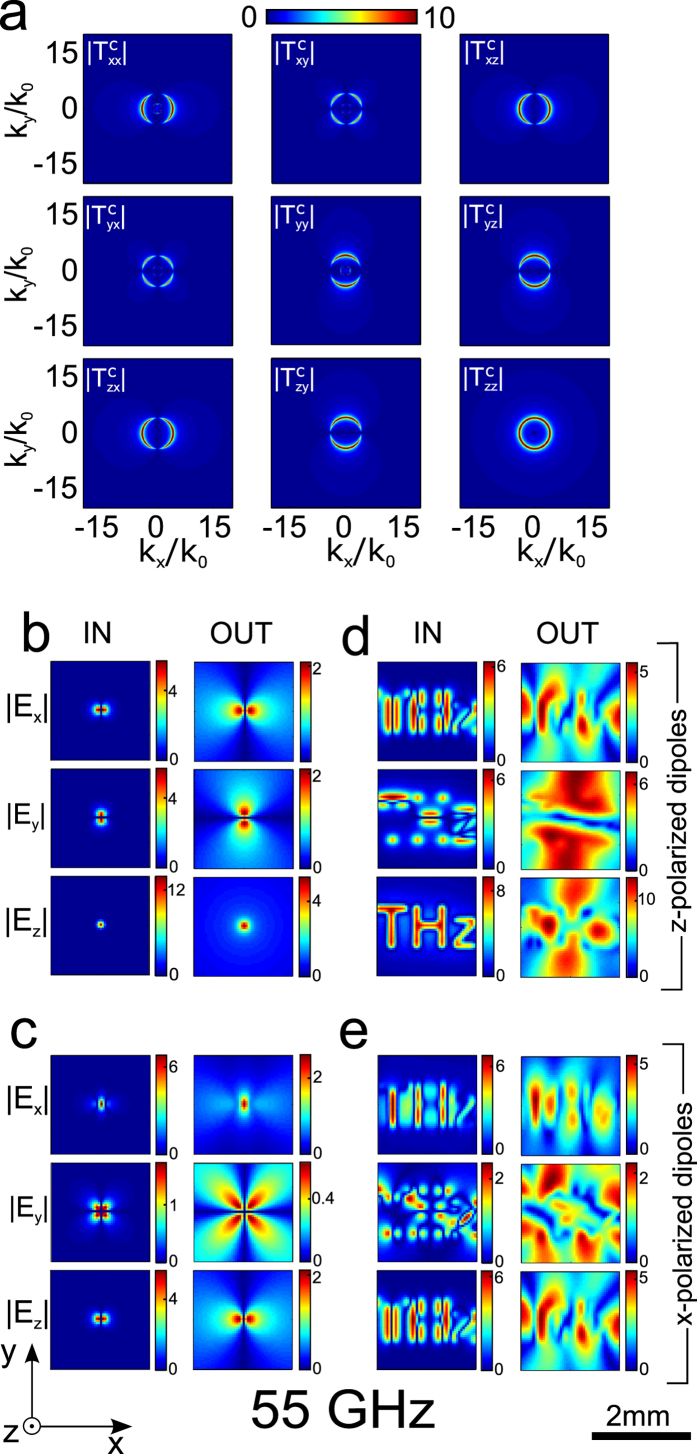
(**a**) Transfer matrix for Cartesian components of the electric field at 55 GHz just below the Fabry-Perot Frequency. (**b,c**) input and output fields for dipoles sources for 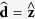 (**b**) and 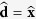 (**c**). (**d,e)** input and output of the letters “THz” formed of dipoles along the same orientation as in (**b,c**) respectively.

**Figure 4:**
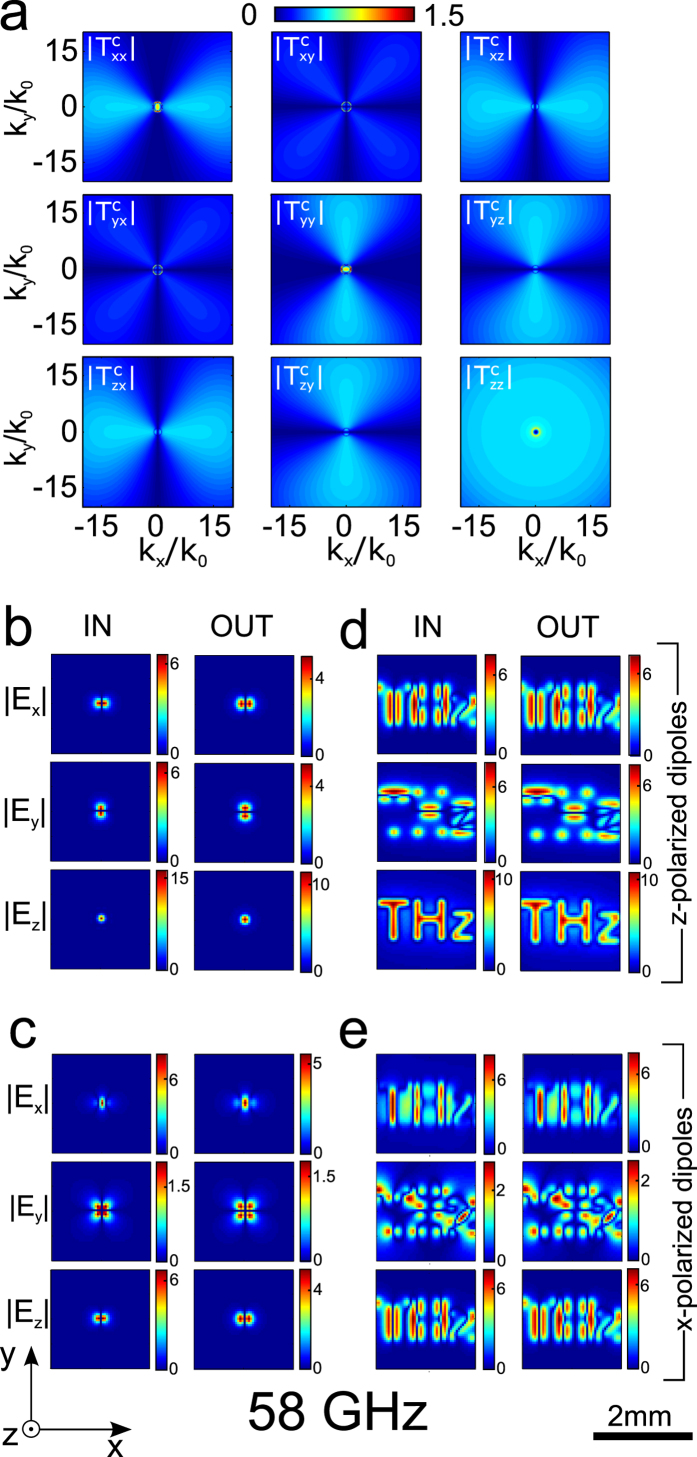
(**a)** Transfer matrix for Cartesian components of the electric field at the Fabry-Perot frequency 58 GHz. (**b,c**) input and output fields for dipoles sources for 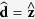 (**b**) and 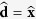 (**c**). (**d,e**): input and output of the letters “THz” formed of dipoles along the same orientation as in (**b,c**) respectively.

To analyse the effect of ordinary and extraordinary waves and the coupling from Cartesian field components, we will now use an illustrative example reproducing experimental conditions of our previous publication on imaging through wire-media fibres for the high GHz and THz spectrum^10^: we consider a finite slab of highly anisotropic medium formed of Zeonex^19^ with length 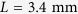 containing an array of indium wires of diameter 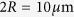 and pitch 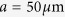. This corresponds to the same parameters presented in Ref. 15. We consider two frequencies: before and at the second Fabry-Perot resonance, corresponding to 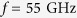 and 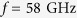, respectively; results are shown in Figs. 3 and 4. Note that imaging is expected to be optimal at frequencies corresponding to Fabry-Perot resonances^12^. For imaging purposes, this medium is well described by a local anisotropic homogenized permittivity tensor with 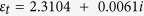 and 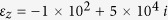 as detailed in the Supplementary Methods 1.2.

The magnitude of all components of the Cartesian transfer matrix 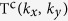 is shown in Figs. 3a and 4a. Note that ordinary waves only propagate for 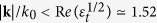 so that at the scale of the figure the main contribution to transmission is from extraordinary waves. Good sub-diffraction imaging is obtained when a broad range of spatial frequencies have near-unity transmission and are in phase.

The shape of 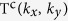 components is a consequence of the transfer function of ordinary and extraordinary waves, and how Cartesian field components couple to them. The former is strongly frequency dependent, while the latter is not. The main difference between Figs. 3a and 4a thus comes from the frequency dependence of the transmission of TE and TM waves.

All transverse spatial frequencies with 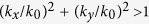 are evanescent in air. As a consequence, high spatial frequency resonant slab modes can exist^20^, leading to larger than unity transmission in this region, as is the case for 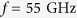: In Fig. 3 all components of T^c^ have a sharp resonance ring due to the existence of a slab mode, which will lead to scrambling of the image. In the absence of losses, transmission would diverge at these spatial frequencies. This resonant ring in 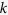-space is only complete for 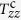, with other components modulated azimuthally, appearing as pairs of two half circles, or four quarter circles. This azimuthal modulation is also present at the Fabry-Perot frequency in Fig. 4, and reflects how much Cartesian components couple with extraordinary waves: taking the example of 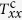, electric field components along 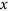 can only couple to and from extraordinary waves for non-zero 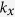, with the strength of coupling given by the relevant dot products in Eqs. (26) and (27).

Figure 4 shows that, as expected, the components of T^c^ are relatively flat and broad functions of 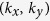 at the FP resonance 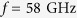, but again are modulated azimuthally by the coupling to and from TM waves for all T^c^ components with an 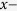 or 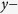 contribution (i.e. all except 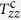). Fig. 4a also clearly shows for 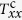 and 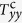 the transmission is higher within the circle of propagation of ordinary waves, with azimuthal dependence inverted compared to transmission outside this circle, showcasing the contribution of ordinary waves to the transmission.

While it comes as no surprise that best imaging will be achieved at the Fabry-Perot resonance, it is worth reflecting on the contributions of different field components to the final image. Clearly the best image would be expected if a purely 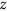-polarized field source could be used, and the 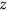 component used for imaging. This however is hardly practical at THz or optical frequencies. Instead, the source field would most often be predominantly transverse, or even more commonly an incoherent superposition of all polarizations. Furthermore, most detectors at optical and THz frequencies are polarization insensitive. For example an object that is predominantly 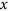-polarized imaged using polarization insensitive near field detectors at the output would result from the contributions of 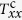, 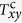 and 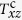. As a consequence high spatial frequencies along the 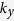 axis would not contribute to images. High spatial resolution would then only be achieved along *x*, with loss of resolution expected in the *y* direction.

### Image of a dipole

We now use the transfer matrix from the above section to calculate the output fields and ensuing point spread function (PSF) of a dipole in two different orientations. We first look at the PSF for each field components separately, as it would be measured by polarization sensitive detectors used in microwave and many THz systems, and then look at the PSF in intensity only, which is relevant for the optical spectrum, and more useful when considering incoherent light sources. We consider a single harmonic electric dipole at the origin. In Cartesian coordinates^21^ the electric field at the slab input A (at 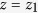) is then given by


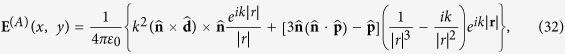


where 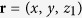, 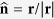, and 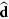 is the unit vector describing the orientation of the dipole oscillation. The 2D-Fourier transform of the dipole field along 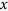 and 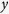 at 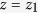 then serves as input field for Eq. (31), and the field in direct space obtained through an inverse Fourier transform of the output fields.

In all below examples 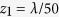 and the image is calculated at the output interface. An illustrative comparison between the electric fields calculated using this method and a commercial finite element solver (COMSOL) at the output of a uniaxial slab, is included in the Supplementary Methods 1.3, validating our approach.

### Dipoles aligned with 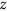 axis

Figures 3 and 4 show the input and output field components in direct space of a single dipole aligned with 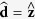 (Figs. 3b and 4b), as well as of an object consisting of 311 dipoles aligned with 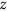 arranged to form the letter “THz” (Figs. 3d and 4d). This is the most favorable case for imaging, as extraordinary waves can be coupled to in all directions through 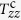. Note the object fits within a 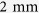 window, imaged at wavelengths around 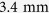.

As expected from the transfer functions, imaging is very good at 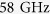 with the image matching the input field almost perfectly. Remarkably, imaging appears to be near perfect for all field components, but this is misleading as the 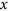 and *y* components of the the output field largely come from the cross coupling 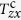 and 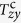 terms.

Images at 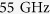 are completely scrambled due to the excitation of the high spatial frequency resonant slab mode.

### Dipoles aligned with 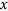 axis

Figures 3 and 4 show the input and output field components in direct space of a single dipole aligned with 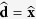 (Figs. 3d and 4d), as well as of an object consisting of 311 dipoles aligned with 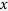 arranged to form the letter “THz”(Figs. 3e and 4e). Again, images at 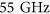 are completely scrambled due to the excitation of the slab mode. At the Fabry-Perot resonance, images are considerably better, and in fact appear almost as good as for *z*-polarized dipoles, but with a slight increase in the spread of the field distribution of the *x* field in the *y* direction. This is consistent with the fact that the *x* dipole can only excite extraordinary waves directly through its dominant *x* field for 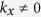, but can still excite extraordinary waves in all directions through its *z* field. If the letters “THz” are harder to read this is mainly because the input field due to different (coherent) *x* aligned dipoles cancel out in numerous places.

**Figure 5:**
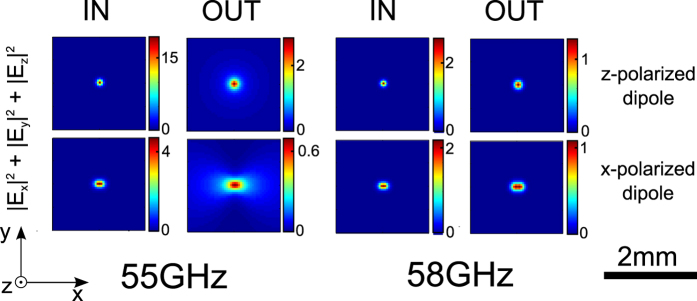
Intensity point spread function for x- and z-polarized dipoles from Figs. 3 and 4.

Figure 5 shows the input and output electric field intensity of the hyperbolic medium slab, for *x*- and *y*-polarized point dipoles at both frequencies. Only at the Fabry-Perot frequency is the PSF narrow. As expected from Fig. 4, *z*-polarized dipoles provide a circularly symmetric PSF, while the PSF of *x*-polarized dipoles is slightly wider and oval shaped.

### Effects of ordinary waves

Aside from perturbation of the sources themselves and reflection and excitation of high-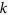 waves studied elsewhere^5^,^11^,^12^,^13^,^14^,^15^, the main causes of artefacts when imaging through a hyperbolic slab are the residual diffraction of the extraordinary waves for finite 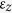, which is negligible in our case, and the contribution of ordinary waves: While extraordinary waves in hyperbolic media are not subject to the diffraction limit, ordinary waves do diffract, which can contribute to direction-dependent blurriness or additional noise. To study this phenomenon, we consider the same hyperbolic medium slab as previously, but with several thicknesses (for which 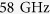 corresponds to higher order FP resonances): the increasing propagation distance will lead to ordinary waves spreading further.

**Figure 6:**
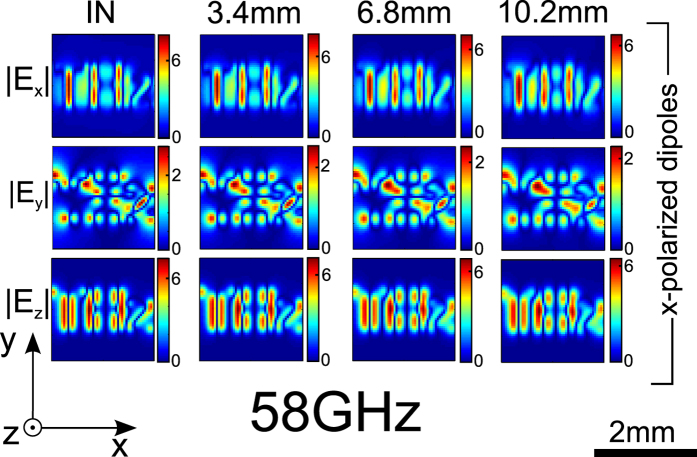
Electric fields for x-polarized “THz” at 58 GHz, for different slab lengths of 3.4 mm, 6.8 mm and 10.2 mm. Ordinary waves barely affect the images, which remain unchanged with increasing propagation distance.

Figure 6 shows the images from the same source *x* aligned dipoles forming the letters “THz” as in Fig. 4e, through slabs of thickness 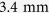, 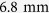 and 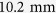. For this polarization, ordinary TE waves are predominantly excited in the *y* direction, and so additional blurriness in the *y* direction is expected. However, this is barely apparent in the images for increased slab thickness. In fact the image used here being entirely contained within half a wavelength, does not have strong low frequency Fourier components. The object thus only weakly excites ordinary waves which would contribute to the distortion.

**Figure 7:**
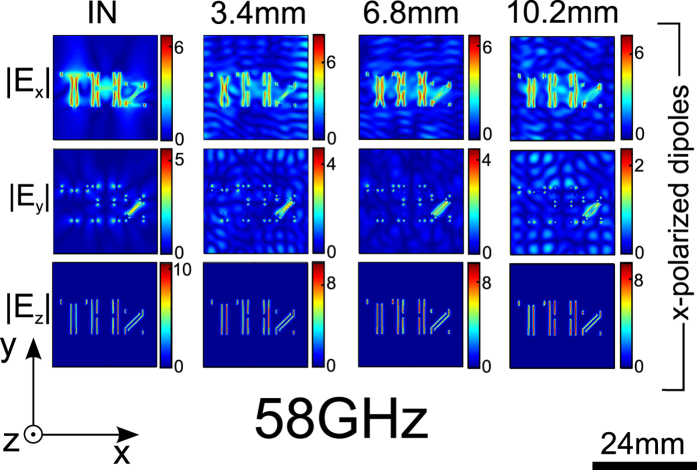
Electric fields for a larger image, x-polarized “THz” at 58 GHz, for different slab lengths of 3.4 mm, 6.8 mm and 10.2 mm. Additional noise is cause by ordinary waves, and is present for the transverse electric field components only.

To back this explanation, Fig. 7 shows images through the same set of thicknesses of a scaled up object, several wavelengths wide (and now consisting of 28017 dipoles). The sources now do have low spatial frequency components exciting ordinary waves. This results in added low-spatial frequency noise even for the shortest propagation distance visible on the *x* and *y* components of the field, and additional distortion with increasing propagation distance. Note that the *z* field components, to which ordinary waves do not contribute, are unaffected by this noise, and remain invariant with propagation distance.

Diffracting ordinary waves from a point source spread with propagation and thus decay in intensity. However, this decay is a simple inverse square dependence. Extraordinary waves, while they do not spread, typically have higher absorption loss and thus stronger exponential decay with distance. Over long enough propagation distances, it is thus expected that relative noise due to ordinary waves increases.

Note that, since for our simulations finite Fourier transforms are used, the source and image are effectively periodic in *x* and *y*, with periodicity of 4.1 cm, filling up the entire *xy* plane. Power emitted by a plane source does not decay due to energy spreading when integrated over an entire plane, and so in our simulations, TE waves, even though they are diffracting, do not decay in power averaged over the unit cell over long distances. However, given the resulting periodicity is much larger than the propagation distance used in our simulations, fields within the unit cell behave as the fields of an individual point source.

## Discussion

We adapted the well known transfer matrix method to study imaging in full two dimensions through hyperbolic media slabs. While we have used a local model only, as is sufficient in many cases^15^, this can readily be extended to non-local models^22^ or used for metal/dielectric multilayer stacks^14^. Propagation through hyperbolic media slabs cause mixing of Cartesian field components, so that field components measured at the output should be interpreted with care. The intensity point spread function depends on the orientation of source dipoles, but not much more than the source field themselves depend on orientation of dipoles.

One of our main motivations for extending the transfer matrix method to full-two dimensional imaging was to study the effect of ordinary waves, in particular in light of experimental results in Ref. 10. It is now clear that for most imaging experiments through hyperbolic media to date, objects were too small to effectively excite ordinary waves. Ordinary waves will start playing a role when sub-diffraction imaging of objects at least a few wavelengths large will be possible, requiring large scale hyperbolic media slabs or hyperlenses. For future devices with such wide field of view, ordinary waves will add noise to the image. This noise can be entirely eliminated if imaging of the *z* component of the field is possible, regardless of illumination polarization, but this is difficult to achieve at optical frequencies.

While this study is limited to planar geometries, a number of conclusions drawn here directly translate to the case of curved geometries and magnifying hyperlenses. To illustrate the different spread of ordinary and extraordinary waves in curved geometries, we calculate in Supplementary Methods 1.4 the point spread function in two polarizations for a cylindrical geometry, at resonance and off-resonance. In the case shown, imaging is also best at radial Fabry-Perot resonance, but the effect of high spatial frequency resonances that are so detrimental in a slab geometry is more limited. Indeed all azimuthal spatial frequencies lower than the diffraction limit on the wide side of hyperlenses (but sub-diffraction on the narrow side) couple to the far field – they are not totally internally reflected, and thus do not lead to strong resonances. When using curved hyperlenses to image in the far field, high spatial frequency resonances are thus naturally filtered out and of little consequence.

Diffracting ordinary waves will also contribute noise in magnifying hyperlenses, but as in the planar case, this should only become a problem if the object to be imaged is larger than a few wavelengths. Perfect imaging (without the noise of ordinary waves) can also be achieved in a magnifying hyperlens if the longitudinal (*i.e.* radial) components of fields can be measured at the output. However since the longitudinal field does not couple to propagating waves and thus has to be measured in the near field, this would largely defeat the purpose of hyperlenses for which imaging is done in the far field. As is the case for planar geometries, Cartesian field components couple differently to ordinary and extraordinary waves in curved geometries, in a way that depends on spatial frequency, so that linearly polarized measurements need to be interpreted with care. For the same reason, separating ordinary from extraordinary waves in the far field cannot simply be done using linear polarizers. Our qualitative conclusions for curved geometries however still require support from a full quantitative investigation of point spread function, field component mixing, separation of ordinary and extraordinary waves etc. in curved geometries. This could also be done by using transfer matrices, but in the somewhat more cumbersome basis of spherical waves.

## Additional Information

**How to cite this article**: Tuniz, A. and Kuhlmey, B. T. Two-dimensional imaging in hyperbolic media - the role of field components and ordinary waves. *Sci. Rep.* **5**, 17690; doi: 10.1038/srep17690 (2015).

## Supplementary Material

Supplementary Information
