# Supplementary material for: Two-dimensional imaging in hyperbolic media–the role of field components and ordinary waves
Source: Sci Rep. 2015 Dec 4;5:17690. doi: 10.1038/srep17690 (PMC4669508; doi:10.1038/srep17690)
Supplement: Supplementary Information [file srep17690-s1.pdf]

# Supplementary Information:
## Two-dimensional imaging in hyperbolic media - the role of field components and ordinary waves

Alessandro Tuniz[1,3] and Boris T. Kuhlmey[1,2*]

[1] Institute of Photonics and Optical Science (IPOS), School of Physics,
The University of Sydney, NSW 2006 Australia

[2] Centre for Ultrahigh bandwidth Devices for Optical Systems (CUDOS),
The University of Sydney, New South Wales 2006, Australia

[3] Leibniz Institute of Photonic Technology (IPHT Jena),
Albert-Einstein-Str. 9, 07745 Jena, Germany

[*] boris.kuhlmey@sydney.edu.au

# 1 Supplementary Methods

## 1.1 S-matrix

The elements of the scattering matrix $S$ (Eq. (22) of the manuscript) are related to the transfer matrix (Eq. (21) of the manuscript) as follows:

$$S_{11} = \frac{T_{24}T_{41} - T_{21}T_{44}}{T_{22}T_{44} - T_{24}T_{42}} \tag{1}$$

$$S_{12} = \frac{T_{44}}{T_{22}T_{44} - T_{24}T_{42}} \tag{2}$$

$$S_{13} = \frac{T_{24}T_{43} - T_{23}T_{44}}{T_{22}T_{44} - T_{24}T_{42}} \tag{3}$$

$$S_{14} = \frac{T_{24}}{T_{24}T_{42} - T_{22}T_{44}} \tag{4}$$

$$S_{21} = T_{11} - \frac{T_{14}(T_{22}T_{41} - T_{21}T_{42}) - T_{12}(T_{24}T_{41} + T_{21}T_{44})}{T_{22}T_{44} - T_{24}T_{42}} \tag{5}$$

$$S_{22} = \frac{T_{12}T_{44} - T_{14}T_{42}}{T_{22}T_{44} - T_{24}T_{42}} \tag{6}$$

$$S_{23} = T_{13} + \frac{T_{14}(T_{23}T_{42} - T_{22}T_{43}) + T_{12}(T_{24}T_{43} - T_{23}T_{44})}{T_{22}T_{44} - T_{24}T_{42}} \tag{7}$$

$$S_{24} = \frac{T_{14}T_{22} - T_{12}T_{24}}{T_{22}T_{44} - T_{24}T_{42}} \tag{8}$$

$$S_{31} = \frac{T_{21}T_{42} - T_{22}T_{41}}{T_{22}T_{44} - T_{24}T_{42}} \tag{9}$$

$$S_{32} = \frac{-T_{42}}{T_{22}T_{44} - T_{24}T_{42}} \tag{10}$$

$$S_{33} = \frac{T_{23}T_{42} - T_{22}T_{43}}{T_{22}T_{44} - T_{24}T_{42}} \tag{11}$$

$$S_{34} = \frac{T_{22}}{T_{22}T_{44} - T_{24}T_{42}} \tag{12}$$

$$S_{41} = T_{31} + \frac{T_{41}(T_{24}T_{32} - T_{22}T_{34}) + T_{21}(T_{34}T_{42} - T_{32}T_{44})}{T_{22}T_{44} - T_{24}T_{42}} \tag{13}$$

$$S_{42} = \frac{T_{32}T_{44} - T_{34}T_{42}}{T_{22}T_{44} - T_{24}T_{42}} \tag{14}$$

$$S_{43} = T_{33} - \frac{T_{23}(T_{32}T_{44} - T_{34}T_{42}) + T_{43}(T_{22}T_{34} - T_{24}T_{32})}{T_{22}T_{44} - T_{24}T_{42}} \tag{15}$$

$$S_{44} = \frac{T_{22}T_{34} - T_{24}T_{32}}{T_{22}T_{44} - T_{24}T_{42}} \tag{16}$$

$$\tag{17}$$

## 1.2 Effective permittivity and local model

For simplicity, the values of relative permittivity for Zeonex (ZNX) and Indium (In) in the narrow range considered in this work (55-58 GHz) are assumed to be non-dispersive, with $\varepsilon_{\text{ZNX}} = 2.3104 + 0.0061i$.[1]

To estimate the permittivity of indium $\varepsilon_{\text{In}}$ we use a Drude model

$$\varepsilon_{\text{In}} = \varepsilon_{\text{In}}^r + i\,\varepsilon_{\text{In}}^i = 1 - \frac{\omega_{\text{In}}^2}{\omega\,(\omega + i\gamma_{\text{In}})}, \tag{18}$$

with indium plasma frequency $\omega_{\text{In}} = 2\pi \times 2.95 \times 10^{15}$ Hz and damping factor $\gamma_{\text{In}} = 2\pi \times 4.04 \times 10^{13}$ Hz. This yields a Drude model fit consistent with that of Ref.[2] Note that the conductivity of indium $\sigma_{\text{In}} = \omega\varepsilon_0\varepsilon_{\text{In}}^i$ at the limit of a constant field yields the measured value for the DC conductivity of indium,

$$\lim_{w\to 0} \sigma_{\text{In}} = \sigma_{\text{In}}^{\text{DC}} = 1.2 \times 10^7\,\Omega^{-1}\text{m}^{-1}. \tag{19}$$

Applying this Drude model to the frequencies considered here yields $\varepsilon_{\text{In}} \approx -5 \times 10^3 + 4 \times 10^6 i$.

In the low terahertz range, it can be shown[3] that this wire medium is very well modeled by an effective permittivity tensor with components (Cf. Eq. (1) of the manuscript) $\varepsilon_t = \varepsilon_h$ and

$$\varepsilon_z(\omega, k_z) = 1 + \frac{1}{\dfrac{\varepsilon_{\text{h}}}{(\varepsilon_m - \varepsilon_{\text{h}}) f_v} - \dfrac{k_0^2\varepsilon_{\text{h}} - k_z^2}{k_p^2}}, \tag{20}$$

where $f_V = \pi R^2/a^2$ is the volumetric filling fraction of the cylindrical wires, $k_0 = \omega/c$, $\varepsilon_h = \varepsilon_{\text{ZNX}}$ and $\varepsilon_m = \varepsilon_{\text{In}}$ are the host and metal permittivities, respectively. The plasma wave vector $k_p$ is related to the geometry of the wire array as follows:

$$k_p^2 = \frac{2\pi/a}{\ln\left(\dfrac{a}{2\pi R}\right) + 0.5257}. \tag{21}$$

Furthermore, the wire medium supports two modes with propagation constants given by,[3]

$$\begin{aligned}(k_z^{(1,2)})^2 \;&=\; \frac{1}{2}\Big\{\varepsilon_t(k_0^2 - k_\parallel^2) + (k_0^2 + k_c^2 - k_p^2) \\ &\pm\; \sqrt{[\varepsilon_t(k_0^2 - k_\parallel^2) - (k_0^2 + k_c^2 - k_p^2)]^2 + 4\varepsilon_t k_\parallel^2 k_p^2}\Big\},\end{aligned} \tag{22}$$

where $k_\parallel = k_x^2 + k_y^2$ and

$$k_c^2 = -\frac{\varepsilon_h k_p^2}{(\varepsilon_m - \varepsilon_h) f_V}. \tag{23}$$

Here, $k_z^{(1)}$ is mostly real, but $k_z^{(2)}$ is mostly imaginary, corresponding to rapidly evanescent waves that do no contribute to imaging.[3] Using the additional boundary conditions for a wire medium,[4] it is then possible to obtain the transmission coefficient $T_\parallel$ for the TM modes of a wire medium slab suspended in air by solving Eq. (19) in Supplementary Ref. 3. Supplementary Fig. S1 shows the transmission amplitude as a function of $k_\parallel$ using this method for the wire medium considered here, at the three frequencies of interest.

This model yields a material-, geometry-, and nonlocal [i.e. $(k_x, k_y)$-dependent] transverse permittivity $\varepsilon_z$ for wire media. Replacing Eq. (22) into Eq. (20) with $k_z = k_z^{(1)}$ yields a spatially dispersive value of

$\varepsilon_z(k_x, k_y)$ which has a *minimum* magnitude corresponding to $\varepsilon_z = -1 \times 10^2 + 5 \times 10^4 i$. Since it has been shown that imaging performance is largely insensitive to $|\varepsilon_z|$ when it is orders of magnitude larger than the transverse component,[5] we thus use a local description for the permittivity tensor of the metamaterial slab with $\varepsilon_t = 2.3104 + 0.0061i$ and $\varepsilon_z = -1 \times 10^2 + 5 \times 10^4 i$. Indeed, the transmission properties of a local metamaterial slab of length 3.4 mm using these parameters are in excellent agreement with the full non-local model, as shown in Supplementary Fig. S1 (dotted lines).

## 1.3 Comparison with finite element method

We compare the fields calculated using the transfer matrix method presented here, with those calculated using a commercially available 3D finite element solver (COMSOL). As an illustrative example, we consider an x-polarized point dipole at a frequency of 300 GHz ($\lambda$ = 1 mm), placed ad a distance of 250 $\mu$m ($\lambda/4$) away from an anisotropic slab with electric permittivity components $\varepsilon_t = 2 + 0.1i$, $\varepsilon_z = 10$ and thickness 0.8 mm. For the 3D finite element calculations we use a slab with dimensions 0.8 mm $\times$ 10 mm $\times$ 10 mm, and a maximum mesh element of 100 $\mu$m due to memory constraints (128 GB RAM). The electric fields at the slab output surface are shown in Supplementary Fig. S2, showing excellent agreement between our method and COMSOL. The transfer matrix method is significantly faster (calculations take minutes instead of hours) and can be performed on any desktop computer.

## 1.4 Curved hyperlens

We now compare the imaging performance of a hyperbolic medium slab with that of a curved magnifying hyperlens. A finite-element simulation of a 3D spherical curved hyperlens is not feasible using the large anisotropic material parameters used throughout this work, due to memory constraints and convergence issues. However, some insight on how our conclusion from planar simulations apply to magnifying hyperlenses can be obtained by perfoming finite-element simulations of a curved geometry in 2D, corresponding to a cylindrical hyperlens. The results are shown in Supplementary Fig. S3. The geometry is composed of a hollow hyperbolic material cylinder of inner diameter 0.2 mm and outer diameter 3.6 mm (corresponding to 3.4 mm radial propagation through the hyperbolic medium and a geometric magnification of 18), radial permittivity $\varepsilon_r = -1 \times 10^2 + 5 \times 10^4 i$, and azimuthal permittivity $\varepsilon_\theta = 2.3104 + 0.0061i$, in an air background. A point source is centred at a distance 50 $\mu$m below the inner wall of the hyperlens. The extraordinary wave behaviour is obtained by exciting an electric dipole along $x$, and plotting the out-of-plane magnetic field $H_y$ (purely transverse-magnetic waves), where propagation and magnification of high spatial frequencies is expected. Results at 55 and 58 GHz are shown in Supplementary Fig. S3(a) and (b), respectively. Note that the magnifying point spread function for extraordinary waves should only weakly depend on frequency, since high azimuthal spatial frequencies are converted to low spatial frequencies upon magnification: Spatial frequencies up to $k_0/36$ on the narrow end can still couple to free space on the wide end, and thus do not lead to strong resonances. As a result, the image scrambling observed in a hyperbolic medium slab at frequencies slightly below Fabry-Perot resonance should be reduced in an equivalent curved structure. Indeed, point source magnification is qualitatively similar at both frequencies; a slightly narrower signal with small additional side-oscillations appears at the output surface of the hyperlens at 55 GHz when compared to 58 GHz [Supplementary Fig. S3(c) and (d)].

Ordinary wave behaviour is analysed using excitation by a magnetic dipole along $x$, and plotting the out-of-plane electric field $E_y$, (purely transverse-electric waves, for which high spatial frequencies do not propagate). In this case, a strongly diffracted field is observed at the output [Supplementary Fig. S3(c) and (d)], suggesting that in a 3D geometry the output signal would be strongly asymmetric. However, to fully comprehend magnifying imaging performance of a curved hyperbolic structure, a complete analysis should be conducted in a spherical basis. This will be the object of future study.

## 2 Supplementary Figures

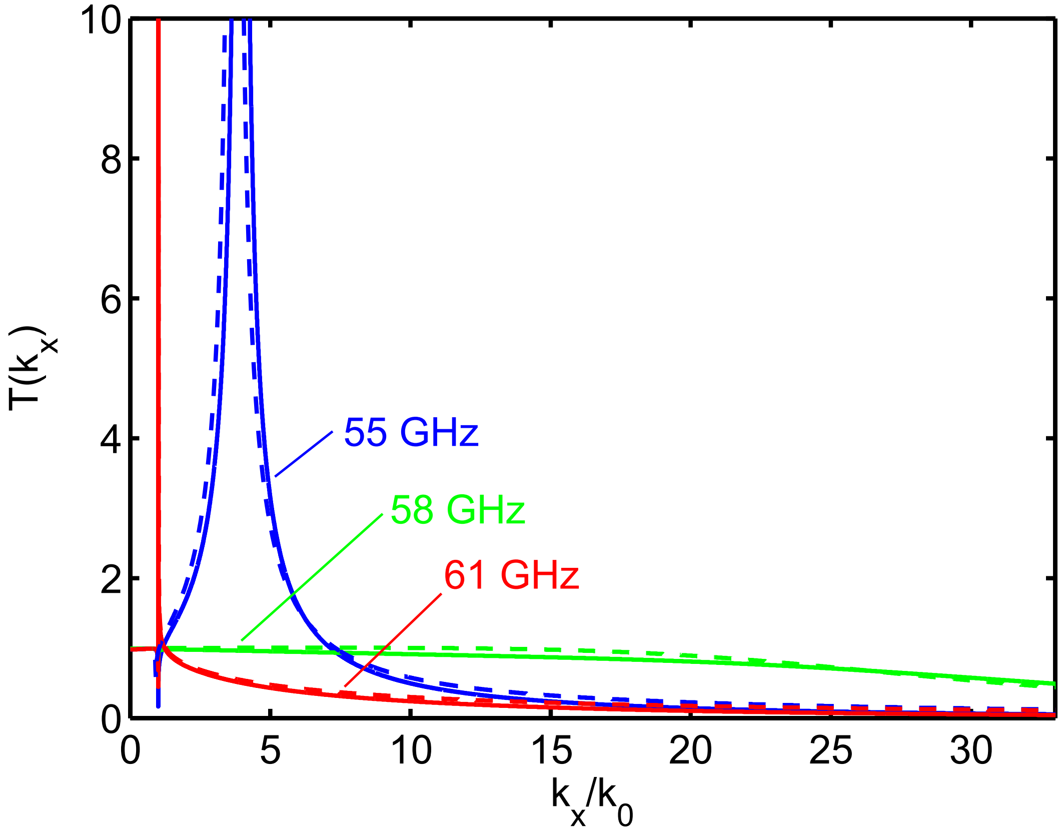


**Supplementary Figure S1: Effective permittivity and local model.** Transmission through the local and nonlocal slabs, as described in the Methods section of the principal manuscript. Solid lines are obtained using the full nonlocal model for wire media. Dashed lines are obtained using a local approximation.

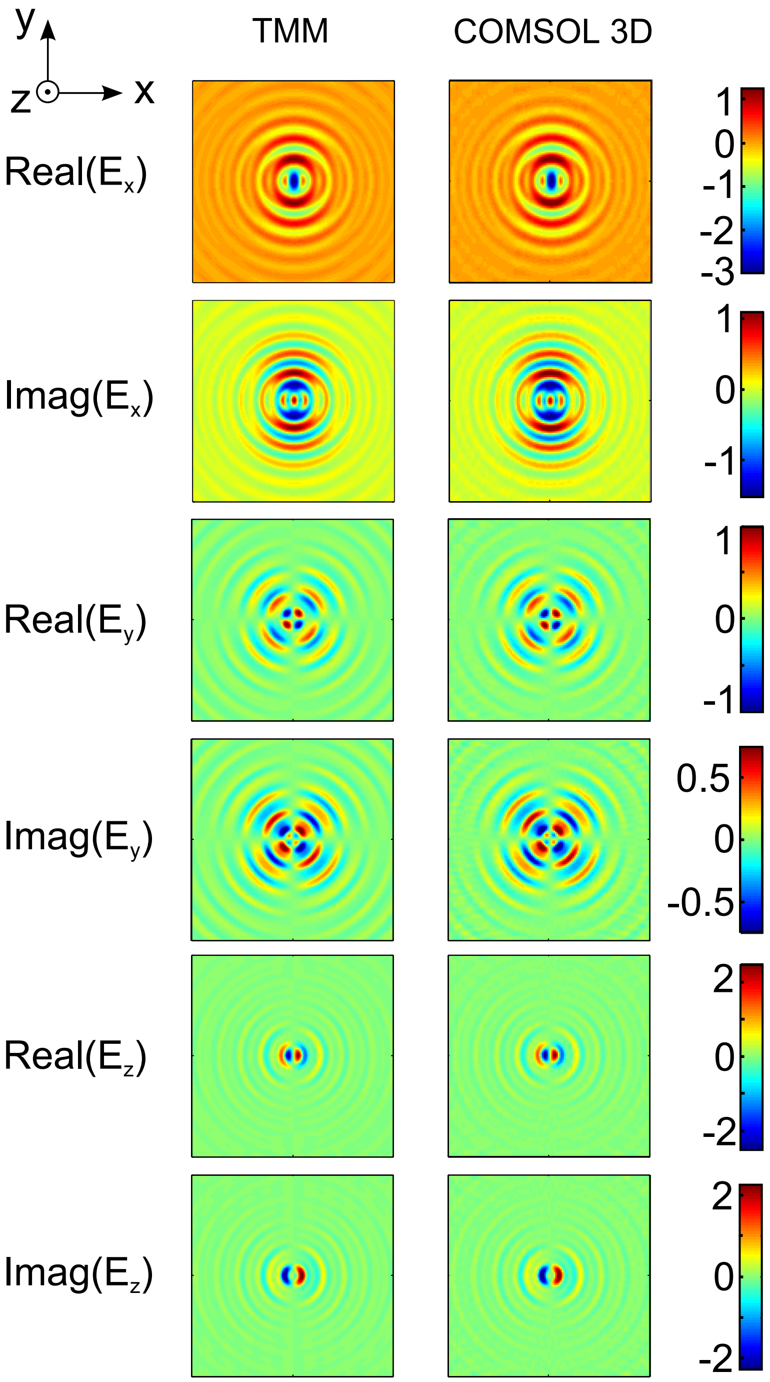


**Supplementary Figure S2: Comparison with finite element method.** Electric field components for x-polarized point dipole in a uniaxial isotropic material, as described in the text. The transfer matrix method presented here shows excellent agreement with the finite element simulation.

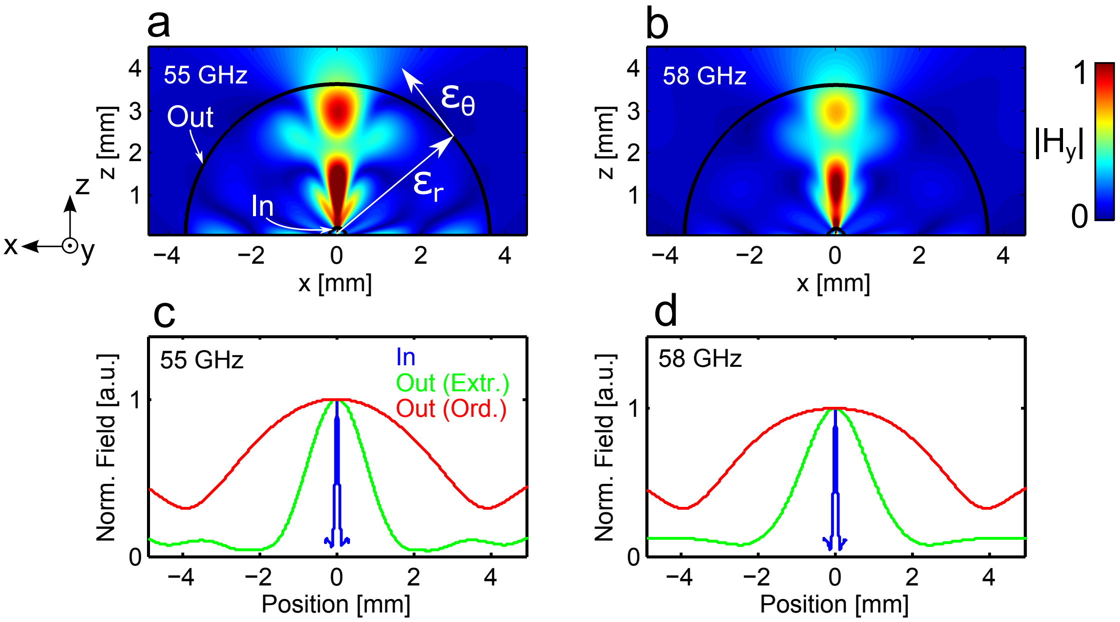


**Supplementary Figure S 3: Curved hyperlens.** Simulations results for 2D cylindrical hyperlens with radial permittivity $\varepsilon_r = -1 \times 10^2 + 5 \times 10^4 i$, and azimuthal permittivity $\varepsilon_\theta = 2.3104 + 0.0061i$, in an air background. A point dipole source is centred at a distance 50 $\mu$m below the inner wall of the hyperlens. The extraordinary field magnitude $|H_y|$ is shown for an $x$-polarized electric dipole at (a) 55 GHz and (b) 58 GHz. Also shown are the field magnitudes along the input (blue) and output surface for extraordinary waves (green) and ordinary waves (red) at (c) 55 and (d) 58 GHz. Curves for ordinary waves correspond to $|E_y|$ for an $x$-polarized magnetic dipole in the same position.

## Supplementary References

[1] J. Anthony, R. Leonhardt, A. Argyros, and M. C. J. Large. Characterization of a microstructured zeonex terahertz fiber. *J. Opt. Soc. Am. B*, 28(5):1013–1018, 2011.

[2] RY Koyama, NV Smith, and WE Spicer. Optical properties of indium. *Phys. Rev. B*, 8:2426–2432, 1973.

[3] M.G. Silveirinha, P.A. Belov, and C.R. Simovski. Subwavelength imaging at infrared frequencies using an array of metallic nanorods. *Phys. Rev. B*, 75(3):035108, 2007.

[4] M.G. Silveirinha. Additional boundary condition for the wire medium. *IEEE T. Antenn. Propag.*, 54(6):1766–1780, 2006.

[5] A. Tuniz, D. Ireland, L. Poladian, A. Argyros, C. M. de Sterke, and B. T Kuhlmey. Imaging performance of finite uniaxial metamaterials with large anisotropy. *Opt. Lett.*, 39(11):3286–3289, 2014.
